# The Role of Probiotics in Skin Health and Related Gut–Skin Axis: A Review

**DOI:** 10.3390/nu15143123

**Published:** 2023-07-13

**Authors:** Ting Gao, Xiaoyu Wang, Yixuan Li, Fazheng Ren

**Affiliations:** Key Laboratory of Precision Nutrition and Food Quality, Key Laboratory of Functional Dairy, Ministry of Education, Beijing Laboratory of Food Quality and Safety, Department of Nutrition and Health, China Agricultural University, Beijing 100083, China; gaotinginging@163.com (T.G.); xy.wang@cau.edu.cn (X.W.); liyixuan@cau.edu.cn (Y.L.)

**Keywords:** skin, probiotics, intestinal microbiota, gut–skin axis

## Abstract

Aging skin, wrinkles, pigmentation, and dryness are problems that plague people, and researchers are working to solve them. Recent studies have shown that intestinal microbiota homeostasis can influence skin health, demonstrating the existence of a gut–skin axis. Recently, improving skin health through probiotic interventions has been proposed, and micro-ecological skin care is becoming a popular concept. By regulating skin health and gut–skin axis interactions, probiotics can be used as potential management tools to suppress and improve skin diseases in multiple ways, including decreasing oxidative stress, suppressing inflammatory responses, and keeping immune effects. The purpose of this paper is to provide a comprehensive review of the application and mechanisms of probiotic-mediated gut microbiota homeostasis in skin care and to offer a theoretical basis for the application of probiotics in skin care.

## 1. Introduction

Skin accounts for about 15 percent of the total body weight of adults, with an average surface area of 1.5–2 m^2^ [1]. One of the main functions of the skin is its use as a mechanical barrier to disease-causing microorganisms and harmful substances; in fact, it could be viewed as one of the host’s vital defenses against infections, as well as the innate and adaptive immune system [2]. Other important features include inhibition of transcutaneous water loss (TEWL), thermoregulation, structural support, and vitamin synthesis, all of which assist in maintaining a healthy host [2,3,4]. 

The quest for beauty never ends. It is often hard to know whether skin issues, including skin pigmentation, skin wrinkles, skin aging, and skin dehydration, occur due to external elements or internal changes. Skin issues have various causes, and investigators are continuously researching safe and efficient skin treatment products to address skin issues. Today, various cosmetic products contain chemicals, including titanium dioxide, which are more or less toxic and may be harmful to an individual’s health [5]. There are also various researchers who use raw materials extracted from herbal medicines as important components in skin treatment products, while they show certain results due to the complexity of herbal ingredients, their influences sometimes fail to meet expectations and their quality still needs to be improved [6,7]. Thus, there is an urgent need to explore safe and efficient ingredients for skin treatment products that can effectively address skin issues. Recently, researchers have suggested that probiotics can be used as an efficient ingredient in cosmetics to address the above-mentioned skin issues to a better effect. In addition, experimental research have indicated that probiotics have no or less toxic influences on hosts and can be better used in the development of skin treatments.

**Figure 1:**
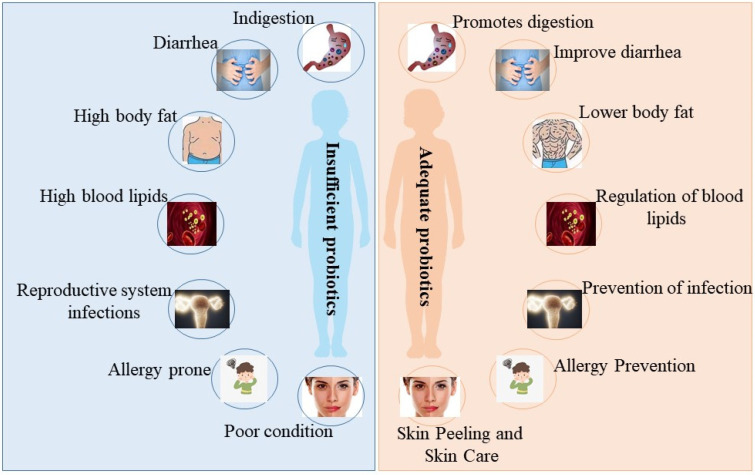
The beneficial effects of probiotics on the organism. When the abundance of probiotics in the organism is insufficient, the organism suffers from the following issues: indigestion, diarrhea, high body fat, high blood lipids, reproductive system infections, allergy prone, and poor skin condition (**left** picture). When the abundance of probiotics in the organism is sufficient, the organism behaves as follows: promotes digestion, improves diarrhea, lowers body fat, regulates blood lipids, prevents reproductive system infections, prevents allergies, and skin peeling and skin care (**right** picture).

Recently, we have become increasingly aware of the powerful effect of probiotics in many fields, and the effects of probiotics in skin care have been increasingly studied and argued by researchers. Probiotics can act through a variety of mechanisms (Figure 1). 

This paper reviews the applications of probiotics in skin care, such as skin whitening, skin moisturizing, skin anti-aging, skin anti-wrinkle, and body odor removal, and their mechanisms, which offers a theory basis for further applications of probiotics in skin care in the future.

Although the applications of topical biologic therapies date back to 1912, when topical use of *Lactobacillus bulgaricus* ameliorated skin problems, including acne and seborrhea, the skin care industry has recently seen a proliferation of topical preparations containing microorganisms [8]. Table 1 lists skin care products containing probiotics sold worldwide.

However, there are many problems with topical probiotics that have not yet been solved. External products cannot be manufactured under sterile status and, therefore, do not require sterility testing. These manufacturers usually include antiseptics to regulate the microorganism’s growth. These antiseptics may influence the viability of probiotic strains and also inadvertently change the microbiota of the receptor [8]. Topical formulations containing probiotics have not yet moved outside the personal care manufacture category; because they assess a high load of colony-forming units, such formulations have difficulty passing the US Food and Drug Administration’s (USFDA) modulatory provisions for microbiota load. Antiseptic effect testing is a vital barrier to measuring these applications. According to the United States Pharmacopeia (USP), topical probiotic preparations for the treatment of acne were tested for microbiota counts. Studies have shown that topical products do not contain “undesirable” amounts of live microorganisms and, therefore, according to the USP [9], do not require less than 1000 colony-forming units (CFU). Meanwhile, since the stratum corneum maintains the skin’s strict natural and protective barrier function, it regulates the absorption of effective substances into the deeper layers of the skin, thus also limiting the choice of treatments [10]. The preparation requirements for topical applications, including live microorganisms, are significantly different from those for products containing only smaller molecules resulting from the need to maintain microbial stabilization. Key factors that are required for microbial control are pH and osmolarity contents, as well as temperature and humidity levels of the storage environment [11].

Topical probiotics have been used to maintain skin health since the beginning of the 20th century, and the last decade has seen a dramatic rise in commercially available topical probiotics [12]. With the increasing popularity of these topical products and the dearth of clinical trials or efficacy studies to establish their clinical efficiency, we are gradually focusing on internal probiotics in treating skin disorders. Since internal probiotics first enter the intestinal tract and inevitably interfere with skin conditions by affecting intestinal homeostasis, this article elaborates on the relationship between probiotics, the intestine and skin, in an attempt to provide potential solutions and clinical value for finding appropriate skin interventions.

## 2. The Different Effects of Probiotics on the Skin (Figure 2)

### 2.1. Skin Whiting

**Figure 2:**
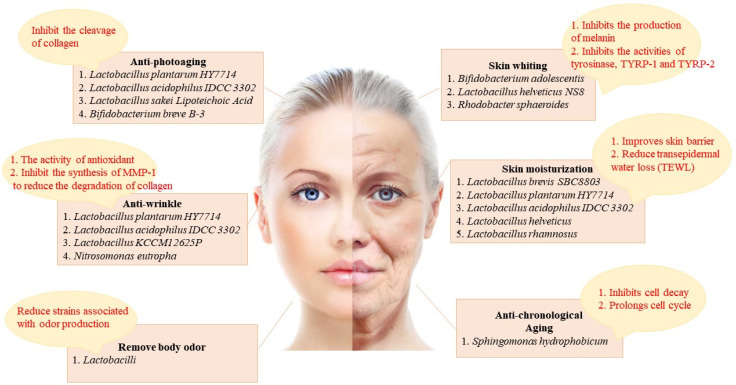
The skin improvement effect of probiotics and its related mechanism. The skin improving effects of probiotics include: anti-photoaging (inhibit the cleavage of collagen), skin whiting (inhibits the production of melanin and inhibits the activities of tyrosinase, TYRP-1 and TYRP-2), anti-wrinkle (the activity of antioxidant and inhibition of the synthesis of matrix metalloproteinase-1 (MMP-1) to reduce the degradation of collagen), skin moisturization (improves skin barrier and reduces TEWL), body odor removal (reduce strains associated with odor production), and anti-chronological aging (inhibits cell decay and prolongs cell cycle).

Recently, there has been an increasing interest in skin lightening, and the focus of brightening products is to decrease melanin content and suppress overproduction pigmentation [13]. Melanin is photoprotective and protects the skin from ultraviolet (UV) radiation, but the overexpression of pigmentation can affect skin tone and even induce various skin disorders, including freckles and melasma [14,15,16]. The process of melanin production involves various enzymes and chemical catalytic reactions [17,18]. There are three main enzymes associated with melanogenesis, containing tyrosinase, tyrosinase-related protein 1 (TYRP-1), and tyrosinase-related protein 2 (TYRP-2), with tyrosinase being the indispensable primary enzyme [19]. A lot of whitening cosmetics can precisely suppress the tyrosinase activity, thus reducing melanin content and achieving a brightening effect. Recently, probiotics have been increasingly used in brightening products, which is tightly associated with their great antagonistic influence on tyrosinase (Figure 2).

A study suggested that the antagonistic influence of *Bifidobacterium adolescentis* culture filtrate on mushroom tyrosinase and tyrosinase activity strengthened with increasing content, thereby reducing the melanin levels in B16F10 cells [20]. According to these studies, *Bifidobacterium adolescentis* culture filtrate could modulate tyrosinase activity via its antioxidant effect, thereby decreasing melanin content and achieving a whitening purpose. Moreover, they also uncovered that lactic acid in *Lactobacillus* can suppress melanin synthesis directly by down-regulating tyrosinase activity and also regulate melanin synthesis by affecting tyrosinase expression or tyrosinase, tyrosine 1 and tyrp-2 to exhibit a brightening effect [21].

Probiotics can decrease melanin content not only by regulating tyrosinase activity but also by other means to achieve a whitening effect. Jingjing Rong et al. indicated the brightening effect of *Lactobacillus helveticus* NS8 fermented milk supernatant (NS8-FS) [22]. Data demonstrated that NS8-FS decreased melanin levels in B16F10 cells by suppressing the activity of tyrosinase and proteins associated with tyrosinase expression. Furthermore, a UV radiation-induced pigmentation model was established in guinea pigs. Masson-Fontana staining and tyrosinase staining tests confirmed that NS8-FS improved skin pigmentation. The potential mechanism by which NS8-FS improved skin pigmentation is the modulation of the Nrf2 activity, which promotes melanogenesis in melanocytes under UV-mediated oxidative stress [23]. Liu et al. explored the inhibitory effect of *Rhodobacter spheroides* (Lysogen™) on melanin synthesis [24]. Research demonstrated that melanin content in B16F10 cells up-regulated after α-MSH supplementation and down-regulated in a dose-dependent manner after Lysogen™ treatment.

### 2.2. Skin Moisturization

There are various reasons for dry skin, such as seasonal alterations, skin barrier damage, and disorderly flaking [25]. Skin hydration is important for health and beauty, and it exerts vital effects on maintaining proper body activity and beauty. Thus, we are continuously searching for molecules that are beneficial for maintain skin moisture. Probiotics can decrease TEWL and improve skin dryness, which can be used to modulate dry skin. Moreover, probiotics can also decrease skin water-loss by modulating skin–barrier function and are good skin moisturizers [26]. 

A study confirmed that the oral supplementation of *Lactobacillus plantarum* HY7714 increased ceramide levels by elevating serine palmitoyltransferase (SPT) mRNA expression and decreasing ceramidase mRNA expression [27]. Ceramides exert an important effect on keeping the structural support of the epidermal barrier and the epidermal hydration [28,29]. Elevated ceramide contents lead to lower TEWL values and up-regulated hydration. Studies used ELISA to detect hyaluronic acid (HA) content and found that the use of acidophilic lactic acid IDCC 3302 exerts a beneficial effect on skin hydration [30,31]. In conclusion, the treatment of *Lactobacillus acidophilus* IDCC 3302 resulted in improved skin dryness and decreased TWEL, thereby up-regulating skin hydration. Hidoko BABA et al. indicated that the administration of *Lactobacillus helveticus*-fermented milk whey (LHMW) led to an obvious reduction in TWEL of intact skin and an increase in skin water content, proving that LHMW milk has a moisturizing effect and is used in cosmetics [32]. 

### 2.3. Skin Barrier Integrity

Damage to the skin barrier function can adversely affect the skin by disrupting the moisture balance on the skin surface [33]. Ye-On Jung et al. demonstrated experimentally that *Lactobacillus rhamnosus* (LR) can effectively improve the skin barrier and can be regarded as a moisturizing skin care product [34]. They used immunofluorescence staining to identify up-regulated expression levels of Claudin-1 and Occludin, two tightly bound molecules and indicated that the stratum corneum of tissues treated with LR lysate was tighter and more organized. Moreover, qPCR results suggested elevated expression levels of loricrin and filaggrin which exert a vital effect on the restoration of skin barrier function [35]. Furthermore, the strengthened skin barrier function was further suggested by reducing sodium dodecyl sulfate (SLS)-induced cytotoxicity and decreasing skin permeability.

### 2.4. Anti-Aging

Chronological aging and photoaging are two primary forms of skin aging [36]. Chronological aging is mainly influenced by internal elements, whereas photoaging is mainly influenced by external elements [37]. These influences are different but have similar regulatory mechanisms, but probiotics have a beneficial effect on both forms of skin aging.

#### 2.4.1. Anti-Chronological Aging

Chronological aging is mainly associated with genetic elements and is a regular physiological process in the human body. As we age, the body ages, and so does the skin, which is characterized by thinning and dryness [38]. Probiotics achieve anti-aging mainly by suppressing cell decay and prolonging the cell cycle. Sandie Gervason et al. indicated that the exclusion of *Sphingomonas hydrophobicum* (SH) could suppress the production of proteins associated with aging, such as P16 and P21, using an immunohistological experiment. P16 and P21 are cell cycle antagonists, which suppress the cell cycle and result in cell aging [39,40]. The production level of P16 and P21 in the experimental group were obviously down-regulated versus the control group without SH extraction. SH extraction was also demonstrated to suppress the SA-β-galactosidase level, which is linked to aging, to improve cell senescence. Moreover, the level of fibrillin-1 and Versican was up-regulated after SH extraction supplementation. Previous research indicated that fibrillin-1 participates in the production of elastic skin fibers [41] and that an up-regulated Versican level can inhibit the apoptosis response of fibroblasts [42], both of which can slow down cell senescence. In the case of SH extraction, it can be used as an anti-aging skin-care product.

#### 2.4.2. Anti-Photoaging

Photoaging is primarily influenced by external environmental elements, such as UV radiation and toxins. These external elements will induce injury to the skin, causing it to lose elasticity, lose moisture, thicken, and become rough and sluggish [43]. Probiotics have a significant influence on the treatment of photoaging, which is primarily achieved by suppressing collagen division.

Research indicated that patients taking *Lactobacillus plantarum* HY7714 had decreased epidermal moisture loss, decreased wrinkle depth, and ameliorated skin gloss and elasticity [44]. Research demonstrated that tyndallized *Lactobacillus acidophilus* IDCC 3302 could restore the reduction in collagen expression after UV irradiation via Western blot analysis [45]. Meanwhile, it was shown that tyndallized *Lactobacillus acidophilus* IDCC 3302 could obviously decrease the contents of MMP-1, MMP-2, and MMP-9 in HaCaT, which were up-regulated due to exposure to UV rays, primarily by suppressing the MAPK signaling pathway. Moreover, tyndallized *Lactobacillus acidophilus* IDCC 3302 can improve the inflammation response by reducing the levels of proinflammatory cytokines, including IL-1β, IL-8, and TNF-α. The above data showed that tyndallized *Lactobacillus acidophilus* IDCC 3302 can inhibit photoaging and ameliorate inflammation responses induced by UV irradiation. You et al. suggested that *Lactobacillus sakei* Lipoteichoic Acid (sLTA) could suppress the phosphorylation of MAPK and further block the MMP-1 synthesis when hosts are exposed to UV rays [46].

### 2.5. Anti-Wrinkle

Wrinkles are induced by atrophy of the skin and repeated contractions of facial muscles underneath [47]. The use of probiotics has been proven to regulate facial wrinkles. The antioxidant activity of probiotics is closely associated with their anti-wrinkle properties. Moreover, MMP-1 synthesis activates the degradation of collagen produced by fibroblasts, resulting in wrinkles on the surface of human skin [48]. Probiotics could suppress MMP-1 synthesis and decrease collagen degradation, resulting in anti-wrinkle properties.

Researchers found that tyndallized *Lactobacillus* KCCM12625P (AL) can suppress the MMP-1 synthesis, thus preventing wrinkle formation. AL can effectively inhibit the formation of facial wrinkles and act as an anti-wrinkle mainly through the above two aspects. 

Hyun Mee Kim et al. found that *Lactobacillus plantarum* HY7714 had a powerful blocking function on UV-induced MMP-1 according to Western blot [49]. Moreover, *Lactobacillus plantarum* HY7714 inhibited the MMP-1 expression and the MMP-2 and MMP-9 activity, which effectively improved the area and depth of wrinkles and exerted a vital effect on wrinkle elimination. A study indicated that tyndallized *Lactobacillus acidophilus* IDCC 3302 could effectively decrease MMP-1, MMP-2, and MMP-9 contents in UV-exposed HaCaT cells measured by ELISA. Therefore, tyndallized Lactobacillus acidophilus IDCC 3302 could decrease wrinkles by suppressing MMPs.

## 3. Presentation of the Gut–Skin Axis

The microbiota of the gut is similar to that of the skin. Various studies have linked inflammatory skin status to intestinal microbiota disorder. The intestinal microbiota affects the body’s immunological function. The immune system defends itself from external pathogenic bacteria. Once the intestinal microbiota is imbalanced, the changed intestinal microbiota may lead to autoimmune and inflammation status not only in the intestines but also in remote organs, including the skin [50]. Various research prove the notion that disorders in the intestinal microbiota could contribute to skin diseases, including acne [51,52], atopic dermatitis [53], psoriasis [54], and rosacea [55]. The immune system appears to mediate the connection between the skin and the intestine. Microbial interactions with the host immune system are important for maintaining skin homeostasis [56]. Thus, a balance between skin and intestines is a logical way to cure many skin disorders. Probiotics exert a crucial effect in improving the microbiota and are a vital therapeutic modality in the cure of inflammatory skin disorders [50]. 

The gut–skin axis suggests a relationship in which the immune properties of the gut microbiota can also influence skin health. The positive modulation of the skin or intestinal microbiota via oral probiotics is regarded as a potential clinical approach to prevent photoaging of the skin. Oral probiotics are a kind of live microbiota that modify the intestinal microbe and can have direct photoprotective influences on special skin cells via regulating immune responses and inflammation factors. In addition, they can increase the serum contents of short-chain fatty acids (SCFAs), which induce a range of immune and inflammatory responses. Oral probiotics have been investigated as a means to directly improve the intestinal microbiota to suppress and cure skin photoaging. In addition, oral probiotics have been used in the treatment of other skin diseases, including atopic dermatitis, acne, rosacea, and psoriasis, by regulating the skin microbiota and gut–skin axis [57,58,59,60,61]. This section discusses the effect of oral probiotics in photoaging and their associated mechanisms.

### 3.1. Improvement of Intestinal Homeostasis by Probiotics

#### 3.1.1. Enhancement of Barrier Function

Probiotics ameliorate gut barrier dysfunction through a variety of potential mechanisms. These mechanisms include upregulation of the expression of mucin proteins, including mucin-type glycoproteins (MUC)1, MUC2, and MUC3, which in turn restrict the movement of bacteria in the mucus, and upregulation of the secretion and expression of antimicrobial peptides and tight junction proteins, including α-defensins, β-defensins and occlusion bands (ZO), to prevent cell proliferation [62].

#### 3.1.2. Suppression of Pathogens

Probiotics compete with pathogenic bacteria or commensals to combine with mucins or epithelial cells and prevent the overgrowth of potentially pathogenic bacteria. Moreover, probiotics offer anti-microbial ingredients, including anti-microbial peptides, SCFA, and bacteriocins, which are associated with inhibiting or killing pathogenic microorganisms. In addition, SCFA, including butyrate, for example, help regulate the expression of occludin and ZO, both of which are associated with the improvement of the epithelial barrier integrity [63]. 

Probiotics also up-regulate the production of IgA in the host gastrointestinal (GI) tract. Secretory IgA defends the intestinal epithelium from colonization and/or invasion via ligation of pathogenic or commensal antigens, induces reverse transcriptional transport of antigens to dendritic cells (DCs), and reduces pro-inflammatory factors [64].

### 3.2. The Pathway of Probiotic-Mediated Intestinal Microbiota Regulating Skin Status (Figure 3)

#### 3.2.1. Immunologic Pathway

**Figure 3:**
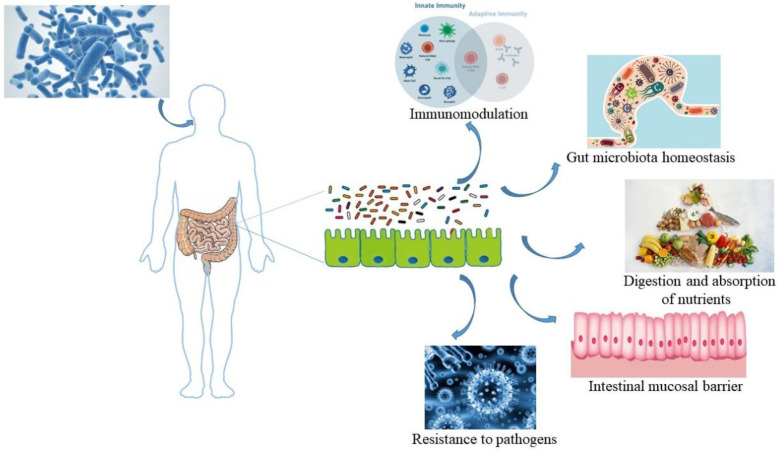
Oral probiotics mediate the beneficial effects of intestinal homeostasis on the organism. After the host ingests probiotics through the oral route, the probiotics enter the intestinal tract and play a role in improving intestinal homeostasis, mainly manifested as: immunomodulation, gut microbiota homeostasis, digestion and absorption of nutrients, and intestinal mucosal barrier.

In terms of immunity to *Staphylococcus aureus*, the most popular bacterial strain influencing atopic dermatitis (AD), an association was indicated between the intestines and skin. *Staphylococcus aureus* is the most common pathogenic bacteria in the skin of AD patients. By contrast, a new birth cohort research showed that colonization with *Staphylococcus aureus* strains exerted a vital effect on preventing the development of AD in infancy, as early responses to *Staphylococcus aureus*, similar to other skin strains, promoted the maturation of the infant’s immune system. *Staphylococcus aureus* strains on the mucosa can play a protective role through immune stimulation [65]. This research supports the speculation that the intestines and skin control the immune environment through the gut microbiota (Figure 3).

Specific intestinal microbiota and their metabolites, including retinoic acid and polysaccharides A separated from *Bacteroides fragilis*, *Facecalibacterium prausnitzii*, and bacterial species attributing to *Clostridium* groups IV and XI, facilitate the accumulation of Tregs and lymphocytes that activate anti-inflammatory responses. In addition, some SCFAs, particularly butyrate, can modulate immune cell activation and apoptosis [66].

The gut microbiome has been studied as an important contributing factor to the immunologic pathway of skin disorders via probiotics. Orally administered probiotics could interact with gastrointestinal mucosa and gut-associated lymphoid tissue (GALT), where more than 70% of immune cells are located [67]. Probiotics interact with epithelial cells, mucosal DC, and macrophages in diverse ways. Depending on the probiotic strain, they can either induce immune activation signaling by producing IL-12, IL-18, and TNF-α or trigger tolerance signaling by stimulating anti-inflammatory cytokines, such as IL-10 and TGF-β [68,69]. In the IL-10/TGF-β-enriched cytokine milieu, DCs and macrophages can enhance the generation of the induced Treg cells that play key roles in maintaining peripheral immune tolerance by balancing the ratio of effector and Treg cells. Apart from probiotics, alterations in the gut microbiome might affect the development of host immune cell function through differences in gut microbiome genes, particularly in infants with AD [70]. 

#### 3.2.2. Metabolite Pathway 

Metabolites originating from intestinal microbiota, including SCFAs, or further supplemented by oral administration, also resolve the link between the intestines and the skin through the microbiota. SCFAs produced by intestinal microbiota including *Akkermansia muciniphila* exert an important effect on the pathology and etiology of AD, which could account for its correlation with the cutaneous immune system. A study showed that linoleic acid and 10-hydroxy-cis-12-octadecenoic acid alleviated AD diseases and controlled the intestinal microbiota in mice [71]. Further, three differential subgroups of the neonatal intestinal microbiota (NGM1–3) and its metabolites have been shown to play a role in early allergic sensitization [72]. Among these three subgroups, NGM3 is associated with multiple allergic sensitizations and was found to be relatively low in content in *Bifidobacterium*, *Ackermannia* and *Fasciola* [65]. For instance, 12,13-dihydroxy-9Zoctadecenoic acid (12,13-DiHome), a metabolite with inflammatory actions on in vitro immune control, was abundant in NGM3. In addition, 12,13-DiHome was up-regulated in the protective layer of casein, the white waxy coating on newborn host skin. These results may suggest the presence of a metabolite pathway in the gut–skin axis [65]. 

#### 3.2.3. Neuroendocrine Pathway 

Similarly to the skin, the lining of the GI is in direct contact with the outside environment, including food and microbes. One of the important roles of the skin and intestine is to suppress the entry of any pathogenic bacteria, and the microorganisms on both organs can be beneficial for the removal of these pathogenic bacteria through immune function, so it is essential to establish a balanced intestinal microbiota in both tissues and keep the appropriate balance for good health. In addition, these two microbiomes can interact with each other via neuroendocrine signaling. This influence can occur through two pathways: direct and indirect [65]. Tryptophan production by gut microbes leading to itchy skin in AD patients is an example of a direct signal. Conversely, γ-aminobutyric acid produced from *Lactobacillus* and *Bifidobacterium* in the intestines inhibits skin itching [65,73]. 

By indirect route, gut microbes control the content of cytokines such as IL-10 and IFN-γ in the blood, which can result in unusual alterations in brain function, leading to anxiety and stress [65]. Cortisol is a representative stress hormone in hosts that can change the permeability and barrier function of the intestinal epithelium by altering the composition of the intestinal microbe [74]. Cortisol can also alter the contents of circulating neuroendocrine molecules, including tryptamine, trimethylamine, and 5-hydroxytryptamine, further enhancing the skin barrier and immunological function [75].

## 4. Probiotics-Mediate Intestinal Microbiota to Improve Skin Disorders (Figure 4)

### 4.1. Acne 

Acne patients have a special skin microbe. Available treatments for acne have various challenges because it injures the mechanical barrier of the skin, thereby drying it out and stimulating it. Research examining the skin–gut axis relationship in acne have shown that treatment with probiotics can improve the immune response beyond the intestines and extend it to the skin [61]. There is increasing evidence that topical probiotics also modulate the skin’s mechanical barrier and generate a secondary up-regulation in antimicrobial peptides. For instance, the lactic acid bacterium *Streptococcus thermophiles* promoted ceramide synthesis when used as a cream, lasting one week in vitro and in vivo [76,77,78]. Ceramides can confine water to the skin, and certain ceramide sphingolipids, including sphingomyelin, have antibacterial activity against *Cutibacterium acnes*, further restoring acne. Through the generation of ceramides, probiotics are used to enhance the skin’s mechanical barrier, which is helpful for acne-affected skin, as ceramides soothe irritated skin [79].

**Figure 4:**
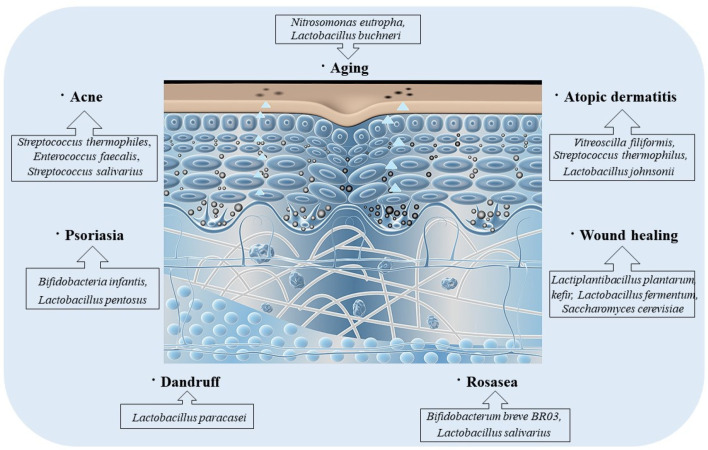
Probiotics can treat skin diseases. Different probiotics can treat different skin diseases, for example, *Nitrosomonas eutropha* and *Lactobacillus buchneri* can improve skin aging; *Streptococcus thermophiles*, *Enterococcus faecalis* and *Streptococcus salivarius* can improve acne; *Vitreoscilla filiformis*, *Streptococcus thermophilus* and *Lactobacillus johnsonii* can improve atopic dermatitis, *Bifidobacteria infantis* and *Lactobacillus pentosus* can improve psoriasia; *Lactiplantibacillus plantarum* kefir, *Lactobacillus fermentum* and *Saccharomyces cerevisiae* can improve wound healing; *Lactobacillus paracasei* can improve dandruff; *Bifidobacterum breve* BR03 and *Lactobacillus salivarius* can improve rosasea.

Thus, probiotics may be used to enhance protective barriers, suppress acne-causing bacteria, decrease pustules, and offer relief from skin irritation in acne patients (Figure 4).

### 4.2. Atopic Dermatitis

AD is mainly caused by a decrease in microbial diversity; as mentioned above, the main microorganism in AD patients is *Staphylococcus aureus*. Various research suggest that oral probiotics may be used as a better option for its treatment [80]. One research showed that *Streptococcus thermophilus* obviously decreased eczema linked to AD and also decreased the severity of symptoms [78]. Another research suggested the validity of the lactic acid bacterium *Streptococcus thermophilus* on the stratum corneum by increasing ceramide levels in the skin [77]. 

In a randomized, double-blind experiment with patients with atopic dermatitis, researchers compared the use of emollients produced by *Lactobaciilus* with the use of regular emollients. Emollients containing Lactobacillus suppressed the extensions of *Staphylococcus aureus*, offered a mechanical barrier, and restored symptoms in patients with AD [81]. An experiment exploring the influence of lotions, including the heat-treated probiotic strain *Lactobacillus johnsonii* NCC on *Staphylococcus aureus* colonization, exhibited a useful effect in clinical symptoms in patients with atopic dermatitis. 

Likewise, other experiments studying the treatment of *Roseomonas mucosa* via supplementation demonstrated an obvious decrease in disease severity, topical steroid needs, and *Staphylococcus aureus burden.* No adverse reactions or complications were reported in this trial [82]. Most of the trials conducted so far have shown that probiotics have a positive effect on patients with atopic dermatitis. 

### 4.3. Psoriasis

Psoriasis is an autoimmune chronic skin disorder that is usually treated with topical emollients and oral immunosuppressants. Few research have included topical probiotics as a treatment for psoriasis. While research has shown that changes in the skin microbiota may help control psoriasis symptoms, oral probiotics have demonstrated therapeutic effects in clinical symptoms in some individuals. However, studies on the validity of oral probiotics in patients with psoriasis are needed to clinically demonstrate the advantages of oral probiotics [60].

### 4.4. Seborrheic Dermatitis

Yeast overgrowth on the scalp and decreased diversity of microbiota leads to dandruff and seborrheic dermatitis. Much research has been conducted to assess the treatment of probiotics in this context. A study of 60 patients exhibited a decrease in erythema, desquamation, and pruritus after the topical application of filamentous Staphylococci [78,83]. Another study revealed that *Vitreoscilla filiformis* lysate induced Treg activity through IL-10 production by dendritic cells [84]. Dandruff, seborrheic dermatitis, and scalp-associated disorders showed beneficial effects after oral supplementation of *Lactobacillus paracasei*. More research is necessary on the local efficacy of probiotics in treating this disease [85].

### 4.5. Rosacea 

Overexpression of TLR2 receptors-induced rosacea arises, resulting in an inflammatory response and changed skin microbiota [86,87]. In addition to doxycycline as an antibiotic, oral probiotics are used to treat scalp rosacea; however, the use of topical probiotics for rosacea treatment has not been explored [88].

## 5. Probiotics That Regulate Skin Physiology (Figure 5)

### 5.1. Nitrobacter

*Nitrobacter* is a nitrifying bacterium that generates nitrate, a molecule that may have positive influences on the host, contained on the skin. Research has shown that dietary nitrates depletion, such as green leafy vegetables, has positive effects, such as increased blood flow to exercising skeletal muscles, decreased exercise oxygen demand, increased exercise tolerance in patients with peripheral arterial disorders, and lower blood pressure. Research has shown that these influences of nitrate depletion are largely the result of NOS-independent increases in NO synthesis and have been indicated to increase cutaneous reflex vasodilation through NOS-independent mechanisms in healthy hosts [89]. Antifungal activity of *Nitrobacter* spp. has been found to defend the skin from dermatophytes [90] and *Staphylococcus aureus* [91], a microbiota that can induce many dermatological infections. The ability of *Nitrobacter* to generate nitrate could also offer nitrate to the skin, which has been proven to conserve the skin’s progenitor cells from UV injury [92,93].

**Figure 5:**
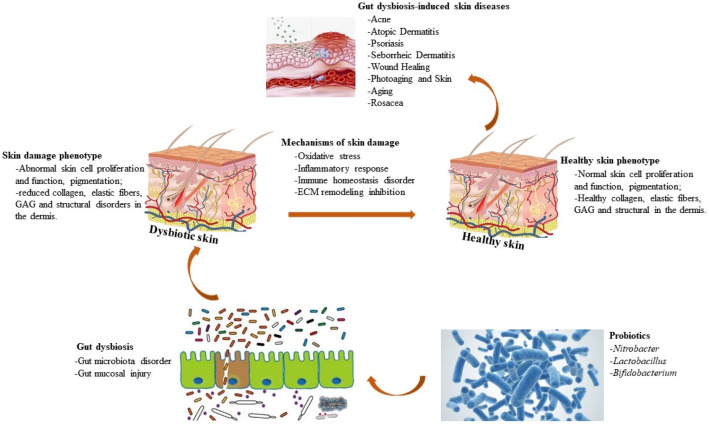
The mechanism of probiotics to improve skin diseases. Probiotics, including *Nitrobacter*, *Lactobacillus* and *Bifidobacterium*, can restore intestinal homeostasis by improving intestinal microbiota disorders and repairing intestinal mucosal damage, and then treat skin damage phenotype, including abnormal skin cell proliferation and function, pigmentation, reduced collagen, elastic fibers, glycosaminoglycan (GAG), and structural disorders in the dermis by inhibiting oxidative stress, inflammation response, immune homeostasis, and extracellular matrix (ECM) remodeling inhibition, ultimately treating skin diseases (acne, atopic dermatitis, psoriasis, seborrheic dermatitis, wound healing, photoaging and aging skin, and rosacea).

### 5.2. Lactobacillus

*Lactobacillus* is the most abundant and diverse genus of lactic acid bacteria [94]. *Lactobacillus* strains have anti-inflammation activity on host keratinocytes and have specific irritation effects on the growth of *Staphylococcus epidermidis* in vitro [95]. *Lactobacillus* also suppresses substance P-caused skin inflammatory responses and promotes the restoration of skin barrier function [96]. A clinical experiment suggested that a six-week oral *Lactobacillus johnsonii* intervention regimen promoted the restoration of skin immune function in UV-caused immunosuppression [97]. In a randomized, double-blind, placebo-controlled experiment program with 20 adults, Fabroccini et al. found that a liquid intervention, including *Lactobacillus rhamnosus,* normalized skin expression of insulin signaling-related genes and improved adult acne [98]. The study showed that tyndalized *Lactobacillus acidophilus*, an oral probiotic, suppressed wrinkle formation resulting from UV irradiation in mice [99]. 

These beneficial influences were due to the reduction in MMPs. Furthermore, Park and Bae indicated in an in vitro study that combined the fermentation of *Lactobacillus* and *Bifidobacterium* to ferment A. koreanum extract inhibited the senescence phenotype of skin fibroblasts [100]. The senescence status is driven by UV- or hydrogen peroxide, while the protection of senescence is partially mediated by the inference of MMP-1 (Figure 5).

### 5.3. Bifidobacterium

Results showed that *Bifidobacterium breve* B-3, as an oral supplementation, obviously inhibited TEWL, skin dryness, changes in epidermal thickening, and improved injury to tight junction structures and basement membranes under excess UV irradiation in mice. *Bifidobacterium breve* B-3 supplementation also inhibited UV-caused production of IL-1β in the skin [101]. 

*Bifidobacterium* and *Lactobacillus* as lyophilized powders in capsules suppress atopic sensitization to general food allergens and decrease the prevalence of atopic eczema in early childhood [102]. In adult patients with atopic dermatitis, *Bifidobacterium bifidum* as an oral supplementation has antipruritic influences linked to up-regulated levels of antipruritic and analgesic metabolite acetonide [103]. In a double-blind, placebo-controlled, randomized trial, ingestion of fermented milk, including *Bifidobacterium breve* and galactooligosaccharides, inhibited reduced levels of stratum corneum hydration, inhibited increased histone l-like protease activity, and decreased serum and urinary phenol contents in healthy adult female volunteers [104].

## 6. Potential Mechanisms of Probiotic-Mediated Regulation of Skin Conditions by the Gut–Skin Axis

### 6.1. Oxidative Stress Level Decreases

The pathological physiology of skin photoaging is tightly related to ROS-caused injury, containing activation of MAPK and NF-κB loop, decrease in MMPs level, and collagen content, resulting in skin photoaging. A study suggested that the partial fermentation of *Agastache rugosa*-fermented extract (ARE-F) with a probiotic *Lactobacillus* promoted UV-caused concentrations of total glutathione and superoxide dismutase activity while down-regulating UV-caused ROS and MMPs levels in UV-treated human keratinocytes cells (HaCaT) keratinocytes [105]. A study indicated that partial *Lactobacillus acidophilus IDCC 3302* defended against UV-caused photodamage in the skin epidermis by promoting the antioxidant capacity of the skin, hydrating cytokines, and inhibiting MMPs synthesis via suppression of MAPK loop [45]. 

Another study suggested that *Lactobacillus acidophilus* KCCM12625 had great antioxidant functions and obviously decreased up-regulated ROS contents in vitro after UV irradiation and improved photodamage induced by oxidative injury [22]. A study suggested that the oral supplementation of *Bifidobacterium breve* Yakult suppressed ROS content and improved UV-caused skin mechanical barrier injury and oxidative stress in vivo [106]. Research showed that a plant extract fermented with *Lactobacillus buchneri* improved the influence of oxidative injury in UV-caused skin photoaging in vitro by up-regulating the type I procollagen content, suppressing elastase synthesis, and up-regulating the level of UV-caused MMPs on HaCaT keratinocytes and dermal fibroblasts [107]. A study suggested that *Limosilactobacillus fermentum* XJC60 could enhance mitochondrial capabilities, decrease ROS content in UV-damaged skin cells, and, therefore, keep the skin status [108]. In addition, new research has indicated antioxidant effect as the primary pathway by which *Lacticaseibacillus rhamnosus* GG (ATCC 53103, LGG) [109] and *Lacticaseibacillus casei* strain Shirota improve skin photoaging [110].

### 6.2. Inflammatory Response Suppression

Up-regulated skin inflammation elements lead to the dysfunction of barrier function, TEWL, incremental permeability of the epidermis, and rapid skin photoaging. Research suggested that oral supplementation of *Bifidobacterium breve* B-3 was effective in decreasing UV-caused IL-1β content in the skin in UV-irradiated mice. As a result, TEWL, skin dryness, and epidermal thickening were inhibited [111,112]. *Lactobacillus acidophilus* IDCC3302, in addition to its antioxidant effects, suppressed MAPK signaling pathway-mediated production of pro-inflammatory factors and decreased UV-radiation-induced skin inflammation [45]. To improve skin photoaging, a study indicated that the oral application of *Lactobacillus reuteri* DSM 17938 showed an anti-inflammation effect which resisted UV-induced IL-6 and IL-8 [113]. 

Keshari et al. suggested that butyrate from a new production of probiotic *Staphylococcus epidermidis* could reduce UV-caused pro-inflammation factor IL-6 factors using SCFA receptors [114]. A study showed that oral oligosaccharides regulated UV-induced inflammatory immune responses and reduced TEWL and sunburn erythema, therefore inhibiting skin photoaging [115].

### 6.3. Immune Homeostasis Maintaining

Many specified probiotics, such as *Lactobacillus paracasei*, regulate immune response to inhibit pathogens [116]. In addition, it inhibits unwanted immunological effects to maintain immune homeostasis against chronic inflammation diseases. It may be due to the modulation of the Tregs number by probiotics. Treg exerts a vital effect on the immunosuppression caused by skin photoaging. *Lactobacillus johnsonii* suppressed UV-caused decrease in epidermal Langerhans cell density and promoted the restoration of skin immune homeostasis after UV-caused immunosuppression. Moreover, probiotics exert different effects under different immune statuses. Under a physiological status, probiotics decrease cytotoxic T cell attack on the skin, up-regulate induction of functional damage to CD8^+^ T cells, and cause the activation of quiescent dendritic cells and the activation and function of all regulatory T cell subsets. A study performed three clinical experiments to analyze the influences of dietary supplements (DS), including *Lactobacillus johnsonii* and nutritional carotenoids, on early UV-caused skin injury [117]. These results suggest that the ingestion of degenerative vertebral slippage has useful influences on the long-term and repetitive influences of UV exposure and is more specific to photoaging. Data showed that oral interventions, including *Bifidobacterium longum* and galacto-oligosaccharides, defend the skin from UV-caused photoaging, resulting in their anti-inflammation and anti-oxidative effects [118]. In addition, they up-regulated serum levels of SCFAs and acetates, which have been shown to up-regulate and activate histone acetylation-dependent skin resident treg.

### 6.4. ECM Remodeling Suppression

ROS contents were up-regulateed after UV exposure, leading to increased MMPs contents, skin collagen protein and elastin protein degradation, and rough, dry and sagging skin. Probiotics can not only directly decrease ROS content but also indirectly regulate MMP levels in the skin, decreasing collagen and elastin protein degradation following UV exposure [119]. Oral *Lactobacillus acidophilus* KCCM12625 could decrease the mRNA level of MMPs after skin photoaging by damaging the AP-1 loop of skin while up-regulating procollagen level and down-regulating collagen protein losses in the dermis [22]. A study indicated that oral *Lactobacillus plantarum HY7714* decreased the overproduction of MMP-13 content and the activities of MMP-2 and MMP-9 in UV-induced cell injuries by suppressing the activation of the JNK/AP-1 loop [49]. Research suggested that oral *Lactobacillus sakei* could suppress theAP-1 expression by inhibiting the MAPK loop to up-regulate collagen in the dermis and improve skin photoaging [46]. Another study demonstrated that extracellular *Lactobacilli* exopolysaccharides (LEPS) can decrease the MMPs level and increase TIMPs [120]. Data showed that LEPS of B9-1 from *Lactobacillus casei* can strengthen the anti-collagenase and anti-elastase functions of the skin and efficiently decrease the breakdown of collagen after UV radiation. A study demonstrated that localized extracts originating *Lactobacillus brucei* fermented plants in kimchi can significantly suppress UV-induced elastase activity and expression of MMPs and enhance the synthesis of type I procollagen. Negari et al. showed that the metabolites from the topical probiotic *Staphylococcus epidermidis* of Cetearyl isononanoate (CIN) as a potential carbon source could restore damaged collagen and cause the synthesis of collagen through phosphorylated extracellular signal-regulated kinase (p-ERK) activation, thereby inhibiting skin photoaging [121].

## 7. Concluding Remarks 

In recent years, significant advances have been made in understanding the composition of skin probiotics and how dysbiosis affects skin health. Topical and internal probiotics in the form of various dermatological formulations are an important part of the treatment of skin conditions. While topical probiotics’ functions and protective nature maintain the skin’s homeostasis, their shortcomings and limitations result in inflammatory skin conditions that are difficult to completely cure via topical probiotics. Several clinical trials are being carried out in order to study the efficacy as well as the adverse effects of internal probiotics formulations for the treatment of conditions such as atopic dermatitis, acne, psoriasis, wound healing, and many other skin problems. We hope that this review forms a contribution to promoting enhanced research activities in the field of internal probiotics as a novel therapeutic approach for the treatment of skin disorders. 

## Figures and Tables

**Table 1:** Skin care products with added probiotics and their effects.

Product	Probiotics	Efficacy
Okana	*Bacillus* bacterial ferment extract	Helps skin retain its firmness and elasticity and keeps it feeling smooth and plump.
Amperna	Unique probiotic complex	Soothes irritated skin and calms redness. Tested on eczema, dermatitis, perioral dermatitis, rosacea, and acne-prone skin.
Cream	1. *Lactobacillus acidophilus* 2. *Lactobacillus rhamnosus*	Anti-photoaging
Elissah Bio P2 Laviol Skin Care	16 types and 35 strains of bacteria including 14 *Bifidobacterium* and *Lactobacilli*.	Strengthens the skin’s barrier against environmental threats and reduces the factors that trigger skin sensitivities, redness, and irritation.
Probiotic Skin Cream Melvory	lactobacilli probiotic (*Lactobacillus* ferment filtrate)	Cleans away the bad bacteria on the skin. For acne-prone or teenage skin.
Andalou Brightening Probiotic + C Renewal Cream	*Bacillus coagulans*	Skin-friendly vegan probiotic microflora enzymatically supports dermal vitality, targeting over-exposed surface cells for a lighter, tighter, brighter looking appearance, and a luminous complexion.
Biossance Squalane + Probiotic Gel	*Lactococcus* ferment lysate	Helps restore the skin’s balance and renew the skin barrier
Neogen Dermalogy Probiotics Double Action	The patented complex of Bifida ferment lysate, *Lactobacillus*, and *Streptococcus thermophilus* ferment	Protects the skin barrier
Elemis Dynamic Resurfacing Facial Pads	*Lactococcus* ferment lysate	Stimulates skin-cell renewal and reinforce the skin barrier
Manyo Factory Bifida Complex Ampoule	Bifida ferment lysate, Bifida ferment filtrate, *Lactobacillus* ferment lysate, and *Lactococcus* ferment lysate	Encourages self-repair of skin, hydrates, replenishes moisture and prevents aging
LaFlore Probiotic Serum Concentrate	*Lactococcus* ferment lysate and live kefir Probiotics (*Hansenula*/*Kloeckera*/ *Lactobacillus*/*Lactococcus*/ *Leuconostoc*/*Pediococcus*/ *Saccharomyces*)	Helps calm and smooth fine lines and wrinkles and boosts the skin’s natural defense system.
Elizabeth Arden Superstart Probiotic Boost Skin Renewal Biocellulose Mask	*Lactococcus* ferment lysate; inactivated strains of *Lactobacillus casei* and *Lactobacillus acidophilus*	Optimizes skin’s microflora and natural defense. Moisturizes and smoothens skin
Dot and Key 72 h hydrating gel and Probiotics	*Saccharomyces* black tea ferment, *Lactobacillus*	Provides hours long moisturization and restores microbiome balance

## Data Availability

Data will be made available on request.

## Abbreviations

TEWL: transcutaneous water loss; MMP-1: matrix metalloproteinase-1; UV: ultraviolet; TYRP-1: tyrosinase-related protein 1; TYRP-2: tyrosinase-related protein 2; SPT: serine palmitoyltransferase; HA: hyaluronic acid; SLS: sodium dodecyl sulfate; SH: *Sphingomonas hydrophobicum*; sLTA: *Lactobacillus sakei* Lipoteichoic Acid; AL: *Lactobacillus* KCCM12625P; SCFAs: short-chain fatty acids; MUC: mucin-type glycoproteins; TJ: tight junction; GI: gastrointestinal; DCs: dendritic cells; AD: matitis; NGM1–3: neonatal intestinal microbiota; GAG: glycosaminoglycan; ECM: extracellular matrix; *ARE-F: Agastache rugosa*-fermented extract; HaCaT: human keratinocytes cells; DS: dietary supplements; *LEPS: Lactobacilli* exopolysaccharides; CIN: Cetearyl isononanoate; CFU: colony forming units; GALT: gut-associated lymphoid tissue.
